# Promoting appropriate medication use by leveraging medical big data

**DOI:** 10.3389/fdgth.2024.1198904

**Published:** 2024-11-07

**Authors:** Linghong Hong, Shiwang Huang, Xiaohai Cai, Zhiming Lin, Yunting Shao, Longbiao Chen, Min Zhao, Chenhui Yang

**Affiliations:** ^1^ Department of Drug Clinical Trial Institution, Xiang'an Hospital of Xiamen University, School of Medicine, Xiamen University, Xiamen, China; ^2^ Fujian Key Laboratory of Sensing and Computing for Smart Cities, School of Informatics, Xiamen University, Xiamen, China; ^3^ Big Data Center, The First Affiliated Hospital of Xiamen University, School of Medicine, Xiamen University, Xiamen, China

**Keywords:** rational use of drugs, appropriate medication, NLP, knowledge graph, transformer

## Abstract

According to World Health Organization statistics, inappropriate medication has become an important factor affecting the safety of rational medication. In the gray area of medical insurance supervision, such as designated drugstores and medical institutions, there are lots of inappropriate medication phenomena regarding “big prescription for minor ailments.” A traditional clinical decision support system is mostly based on established rules to regulate inappropriate prescriptions, which are not suitable for clinical environments and require intelligent review. In this study, we model the complex relationships between patients, diseases, and drugs based on medical big data to promote appropriate medication use. More specifically, we first construct the medication knowledge graph based on the historical prescription big data of tertiary hospitals and medical text data. Second, based on the medication knowledge graph, we employ a Gaussian mixture model to group patient population representation as physiological features. For diagnostic features, we employ pre-training word vector Bidirectional Encoder Representations from Transformers to enhance the semantic representation between diagnoses. In addition, to reduce adverse drug interactions caused by drug combinations, we employ a graph convolution network to transform drug interaction information into drug interaction features. Finally, we employ the sequence generation model to learn the complex relationships between patients, diseases, and drugs and provide an appropriate medication evaluation for doctor prescriptions in small hospitals from two aspects: drug list and medication course of treatment. In this study, we utilize the MIMIC III dataset alongside data from a tertiary hospital in Fujian Province to validate our model. The results show that our method is more effective than other baseline methods in the accuracy of the medication regimen prediction of rational medication. In addition, it achieved high accuracy in the appropriate medication detection of prescription in small hospitals.

## 1 Introduction

The rational use of medicines is safe, effective, affordable, and appropriate for treating or curing the patient (1). The inappropriate use of medicines is a major problem worldwide. The World Health Organization (WHO) estimates that more than half of all medicines are prescribed, dispensed, or sold inappropriately and that half of all patients fail to take them correctly (2). In addition, in the gray area of medical insurance supervision, such as designated pharmacies and medical institutions, there may be “big prescription for minor ailments” healthcare fraud (3, 4). Inappropriate drug use behaviors such as the overuse, underuse, or misuse of medicines not only waste medical resources but also lead to significant patient harm in terms of medication errors (MEs) and adverse drug events (ADEs) (1). The WHO is committed to promoting the *rational use of medicines* for clinical physicians and pharmacists to ensure that “patients receive the appropriate medicines, in doses that meet their own individual requirements, for an adequate period of time” (1).

One of the key challenges in the rational use of medicines is appropriate medication use. Compared with the safety, effectiveness, and economics of rational drug use, the evaluation of the appropriate use of medicines is more complicated, involving hyper-medication, under-medication, and inappropriate medication.

To address these issues, experienced investigators are assigned to hospitals to manage Medicare fraud detection. However, this method becomes time-consuming and inefficient due to the large amount of data collection. With the advent of the big data era, healthcare big data analysis can offer predictive modeling, clinical decision support, disease or safety monitoring, and other capabilities for public healthcare (5). Improvements in data mining and deep learning tools have turned attention to automated systems for fraud detection. Several deep learning-based clinical decision support systems (CDSSs) have been developed and deployed in hospitals to reduce the incidence of improper drug use.

Leveraging the application of knowledge graph construction and sequence model generation makes medication decision-making in the field of pharmacy more scientifically rational (6). Healthcare practitioners can gain a comprehensive understanding of the interrelationships between medications, and sequence generation can optimize medication plans based on patients’ medical histories, symptoms, and physiological data. For example, in the safety of rational medicines, Shao et al. (7) construct a probabilistic probability model of massive prescription data based on a knowledge graph to evaluate the risk of a drug combination by a graph search algorithm. In the rational use of medicines, Shang et al. (8) jointly model the longitudinal patient records as an electronic health record (EHR) graph and the drug knowledge base as a drug–drug interaction (DDI) graph through the generation of sequence models that train end-to-end to provide effective and safe medication recommendations. Based on the experimental results on real-world EHR, GAMENet outperformed all baselines in DDI rate reduction (8). After analyzing a large number of medical records, the diagnosis-related groups (DRGs) payment system (9) based on disease type has been launched by The National Medical Insurance Administration to specify uniform drug delivery rules and prevent excessive medical treatment. However, the single and rigid pharmaceutical rules cannot achieve more accurate personal medication, which also poses a major challenge to the promotion of DRGs (10). To address this issue, we need a more flexible and intelligent method for the evaluation of appropriate medication.

Fortunately, with the emergence of medical consortia and the sinking of medical resources, the professional prescription experience of tertiary hospitals can be accessed, providing us with new perspectives to address the problems existing in designated pharmacies and medical institutions. Therefore, we integrate the clinical medication experience of tertiary hospitals and medical knowledge and transfer the learned knowledge to small hospitals and clinics so that their prescriptions are more in line with professional standards. To achieve these goals, we need to address the following issues.

Owing to the large individual differences in patients, such as being children, adults, or older, and differences in their liver and kidney functions, nervous system, and other physiological characteristics, the same diagnosis may lead to different treatment regimens. The majority of drugs are administered based on the patient’s age or weight (mg/kg) (11). Therefore, to remedy the case with greater precision, we need to consider the individualized use of medicines.

Since the relationship between disease and symptoms is not a simple one-to-one relationship, the occurrence of a single disease may cause the simultaneous occurrence of multiple symptoms (12); therefore, doctors must treat patients through the combination of multiple drugs. Multimorbidity (13) is becoming more common and is a growing global challenge. Therefore, it is a challenge for us to address the complex relationship between disease and drug use.

The increase in drug species shows that the compatibility relationships between drugs are more complicated. In addition, there would be more drug overuse and abuse in the case of “big prescription for minor ailments,” and polypharmacy may increase drug side effects and even more adverse drug–drug interactions (14–16). Therefore, DDIs should be taken into account when evaluating the appropriateness of rational drug use to reduce adverse reactions associated with combined drug prescriptions.

In the preceding discussion, we delved extensively into the interconnections among patient characteristics, diagnosis, and prescription medications. However, in real-world scenarios, the relationships among these three components are even more intricately intertwined. A patient’s individual attributes, such as gender, age, and medical history, exert a significant influence on the susceptibility to diseases, progression of the illness, and response to treatment (11). Diverse patient characteristics may give rise to distinct pathophysiological processes, thereby impacting the selection of diagnostic and therapeutic strategies for the ailment. This, in turn, substantially affects the physician’s ability to accurately diagnose the condition and formulate an effective treatment regimen (17). The precision of the diagnosis is pivotal in devising a successful therapeutic plan. Simultaneously, the choice of medications must take patient-specific features into account, including age, gender, baseline health status, and potential interactions with other medications (18). Furthermore, patient attributes can also influence the individual’s response to and tolerance of pharmaceuticals; for instance, certain medications may be metabolized at a slower rate in older patients, necessitating dose adjustments to avert adverse reactions. To sum up, these three elements intricately intertwine, paving the way for patients to access optimal treatment pathways and furnish a robust foundation for scientifically sound medication recommendations.

To address the above issues, in this study, we propose a regulatory framework of rational drug use based on medical consortia and big data through mining the clinical experience of prescription big data and medical knowledge of drug instructions. To be specific, we first extract information from the big data of prescription of tertiary hospitals and medical text data, and establish the medication knowledge graph based on the extracted information. Second, based on the medication knowledge graph, we extract physiological, diagnostic, and drug interaction features through feature enhancement. Finally, we construct the sequence generation model to solve the complex relationship between patients, diseases, and drugs and then evaluate the appropriate medication prescribed by doctors in small hospitals using the model learned from a tertiary hospital.

In conclusion, the contribution of this study is as follows:

1. To the best of our knowledge, this is the first study on the data-driven evaluation of appropriate medication use. By utilizing extensive prescription data from tertiary hospitals and integrating medical text information, we provide a practical tool for assessing and improving prescription practices in small hospitals, focusing on drug selection and treatment courses.
2. We propose a data-driven experience extraction of clinical rational drug use and an appropriate medication evaluation framework based on advanced deep learning techniques. This approach facilitates the transfer of rational drug use practices from tertiary hospitals to primary care settings, thereby ensuring safer and more effective medication management in these environments.
3. We evaluate the proposed framework with two medical record datasets: Medical Information Mart for Intensive Care III (MIMIC_III) and real-world prescription big data collected from tertiary hospitals. Results show that our method has more accurate medication regimen prediction ability and consistently outperforms other baselines. In addition, it has achieved high accuracy in the appropriate medication detection of prescription in small hospitals.
4. Our research utilizes medical big data to improve medication use practices by addressing important public health challenges, such as MEs and ADEs. Through the analysis of data on prescriptions and patient outcomes, our study aims to support the development of drug safety monitoring and medication management practices.
5. Furthermore, the methodologies and findings of our study have profound implications for clinical trials. Our data-driven approach allows for a better understanding of drug efficacy and safety across diverse patient demographics, aiding in the design and evaluation of clinical trials. This is particularly crucial in trials that aim to tailor medical treatments to individual patient needs, a cornerstone of personalized medicine.

The remainder of this paper is organized as follows. We first elaborate on the proposed framework in Section 2, and then present our experiments in Section 3. Finally, a comprehensive summary of our work is encapsulated in Section 4.

## 2 Methods

**Figure 1:**
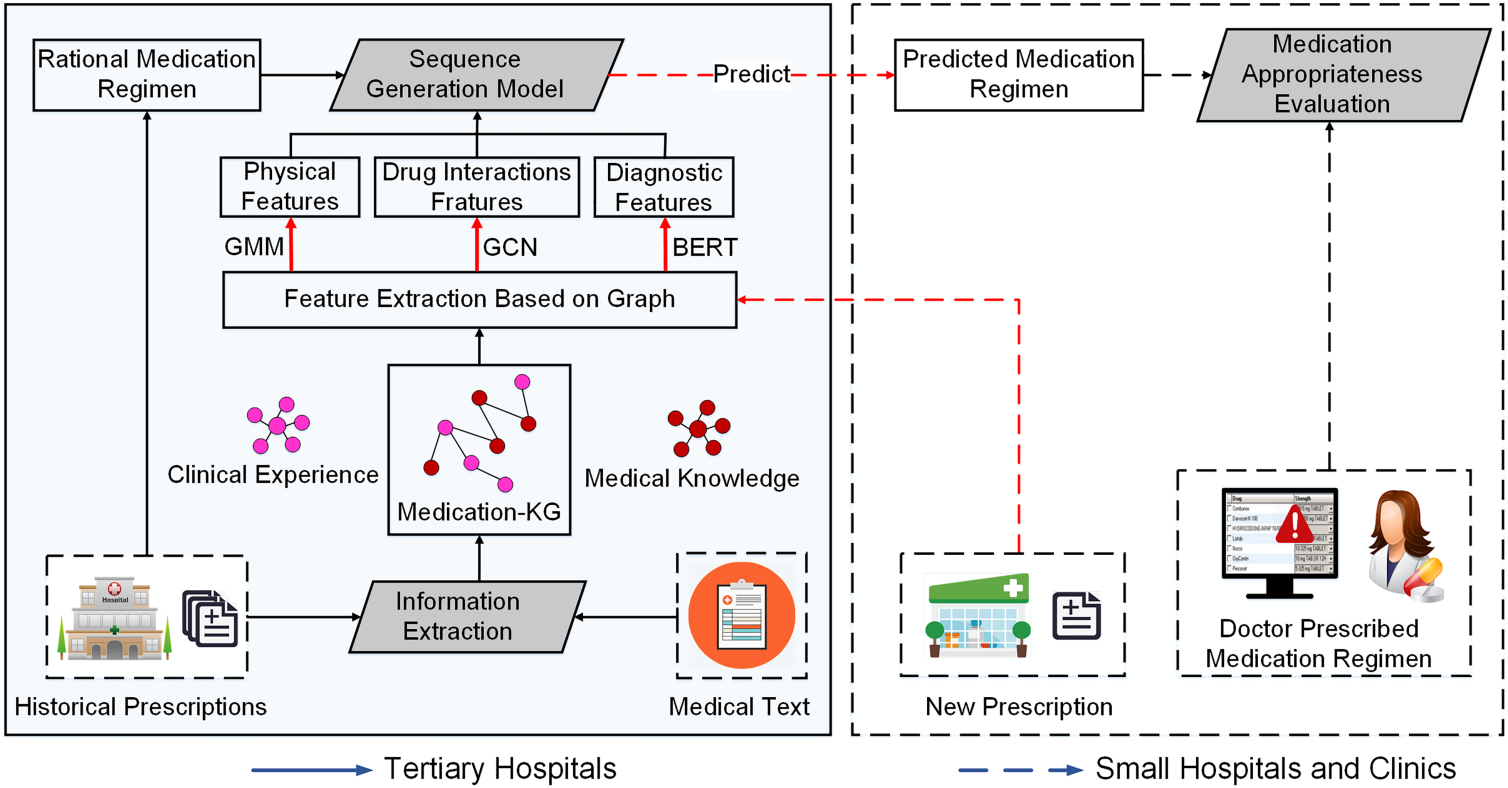
An overview of the proposed framework.

We propose a framework for the experience extraction of clinical rational drug use and appropriate index evaluation, as illustrated in Figure 1. In the *medication knowledge graph construction* stage, we first extract drug triads from historical prescriptions and medical text data, then establish a patient–disease–drug knowledge graph. In the *modeling* phase, we first employ a Gaussian mixture model (GMM) (19) to group patient population representation as physiological features, based on the four physiological variables of gender, age, height, and weight. Second, we transform patients’ diagnostic information into word vectors as diagnostic features through pre-training word vector Bidirectional Encoder Representations from Transformers (BERT) (20) to enhance the semantic representation between diagnoses. Third, to reduce adverse drug interactions caused by drug combinations, we employ a graph convolution network (GCN) (21) to transform drug interaction information into drug interaction features. Finally, we exploit the medication regimen from historical prescription data to train a sequence generation model. In the *analysis* stage, given a new prescription from small hospitals or clinics, we use the trained model to predict the rational medication regimen for the prescription, and provide an evaluation of appropriate drug use in terms of the drug list and medication course of treatment to physicians and pharmacists in small hospitals. We elaborate the details of the key components in the following.

### 2.1 Medication knowledge graph construction

In this section, our objective is to construct a medication knowledge graph to model medication rules for co-prescription in big data. However, relying only on historical prescription data is not enough to simulate the comprehensive medication rules, because adverse drug reactions (ADRs) may not be reflected in clinical practice. Therefore, we also incorporate the drug interaction information extracted from the drug instructions as a supplement. First, owing to the large amount of non-(semi-structured) data in historical prescription big data and drug instructions, we need to transform these data into structured triplet data. Second, we build the clinical experience edges and medical knowledge edges based on the structured data of historical prescriptions and drug instruction. Finally, we construct our medication knowledge graph according to two kinds of edges. We elaborate the details as follows.

#### 2.1.1 Information extraction

In this step, to extract drug entities and relationships, we modeled the problem as an information extraction task in natural language processing (NLP) and solved it using information extraction technology. First, for historical prescription data, we transformed semi-structured disease–drug–diagnosis information into structured clinical triples to achieve a complete delineation of clinical experience. Then, for auxiliary medical text data, we extracted medical knowledge triples to supplement the medication knowledge graph. The information extract details are elaborated as follows.

*Clinical experience extraction.* In this step, we extracted clinical experience based on the collected prescription big data. The historical prescription data mainly include the prescription number, patient’s age, height, weight, and other personal signs, the diagnosis of disease, drugs, and their course of treatment, and other information. To better show the clinical medication experience, we established explicit attributes of entities and implicit triple relationships between entities according to medication knowledge.

Specifically, we first extract different entities in the prescription, including patients, diseases, and drugs. Then, we regard physiological characteristics such as gender, age, height, and weight as the attributes of patient entity. In addition, if there is a diagnosis on the prescription that is associated with a pregnant woman, such as at 14 weeks of gestation, the patient will be given the role of pregnant woman. Furthermore, we construct implicit relationships between different entities based on prescriptions, such as the relationship between the patient and the drug, the relationship between the patient and the disease, and the relationship between the diagnosis and the disease. Finally, we iterate over each prescription and use a triple to represent all the entities in the prescription and their relationships, such as “Influenza–Prescribe–Ribavirin Spray,” etc.

**Figure 2:**
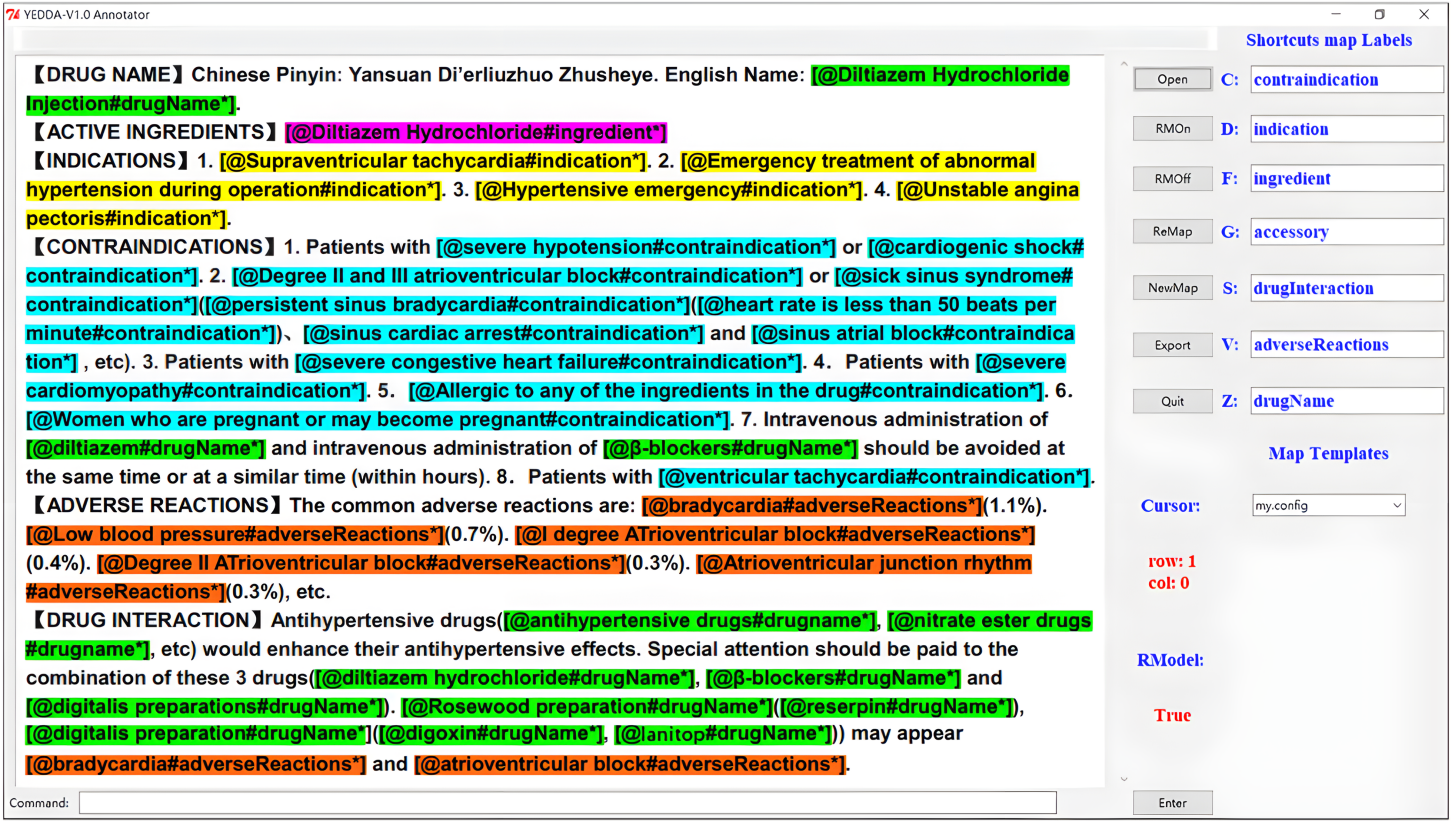
We use the YEDDA tool to label the entities in the drug instructions, and the labeled entities include the drug names, diseases, ingredients, indications, adverse reactions, and contraindications.

**Figure 3:**
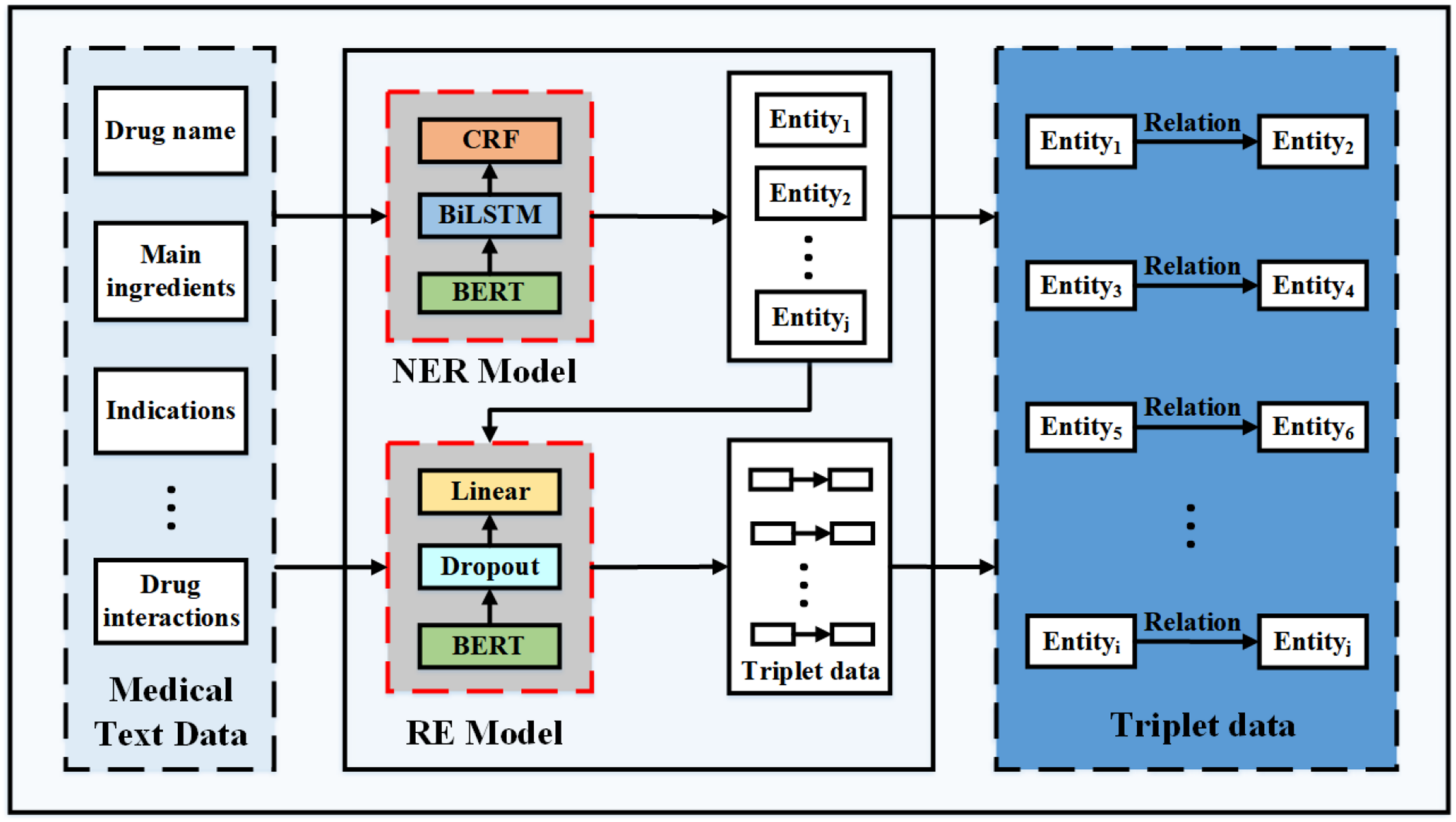
The medical knowledge extraction framework.

*Medical knowledge extraction.* In this step, we extract medical knowledge based on the collected dataset of drug instructions. As there is an implicit regional structure in each of the drug instructions, as shown on the left in Figure 2, we first divide a part of the collected drug instruction data into blocks to extract the required structured information, such as drug name, main ingredients, indications, contraindications, adverse reactions, precautions, and drug interactions. Second, we manually label the pre-processed dataset based on the open source labeling tool YEDDA, as shown in Figure 2. In addition, the labeled entity includes but is not limited to the drug names, diseases, ingredients, indications, adverse reactions, and contraindications. Third, we also marked another dataset in the format of (text, entity, relationship, entity) on the module data of annotated notes and drug interaction to extract drug interactions. According to the harm degree of drug interaction to the human body, the relationship fields of drug interaction are divided into four categories, which are beneficial, no effect, unknown, and harmful. Finally, we model the medical knowledge extraction problem as named entity recognition and relation extraction tasks in NLP to extract medical triplet information, as shown in Figure 3.

Specifically, we first train the Bert-BilSTM-CRF model to recognize medical entities, including drug, ingredient, disease, indication, and contraindication. The Bert-Bi-LSTM-CRF model was proven to outperform all other models in the NLP of Chinese electronic health documents (22). Second, to extract the relationships between entities, such as drug interactions, we construct the relation extraction model (RE model): BertModel + Dropout + Linear. Finally, we employ the trained entity recognition model to extract medical entities. In addition, for drug interaction data, we identify the relationships based on the extracted entities. There are two approaches to form medical triplet data. The first method is to take the drug name and other entities as the first entity and the second entity, respectively, and label as the relation, such as “Ribavirin spray–Ingredient–Ribavirin.” The second method is to use the triplet data extracted from the RE model, such as “Cefoperazone sodium for injection–Contraindication–Amikacin.”

#### 2.1.2 Graph node and edge construction

**Figure 4:**
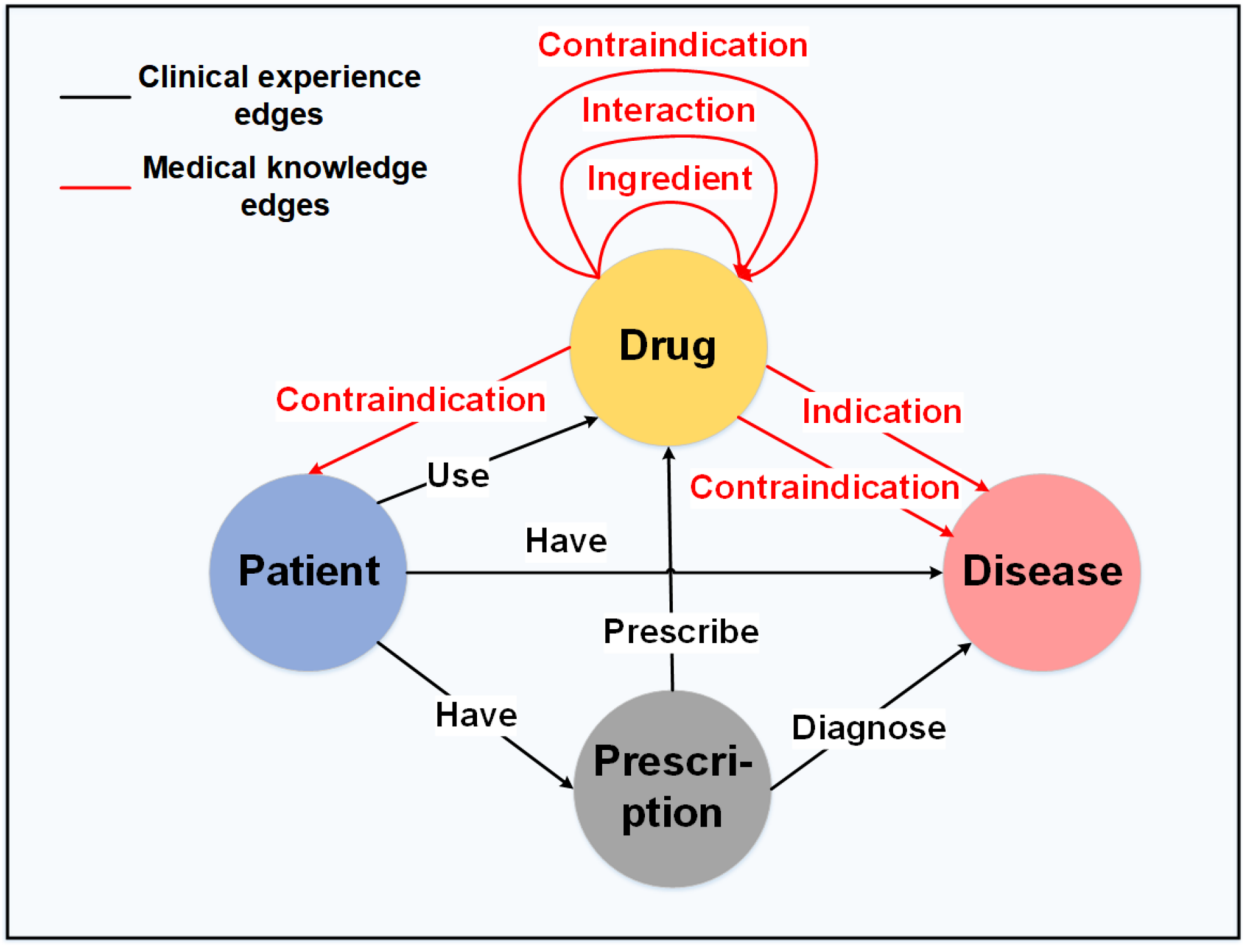
The structure of the medication knowledge graph.

To construct the medication knowledge graph G=(E,R), we define entities (E) and the relationships (R) between them, as illustrated in Figure 4. In graph theory, the triple Q is defined as the set of $\left(e_{i},r,e_{j}\right)$, where $e_{i}$ and $e_{j}$ denote two different entities, and r denotes the relationship between node $e_{i}$ and node $e_{j}$. As shown in Figure 4, the black edge sets represent the clinical experience edges $R_{a}$, and the red edge sets represent the medical knowledge edges $R_{b}$. The detailed construction information can be found in the Appendix.

### 2.2 Drug recommendation model based on knowledge graph

In this section, our objective is to model the complex relationships between patients, diagnoses, and drugs based on the medication knowledge graph constructed in the previous phase. As there are many prescription features in the medication knowledge graph, we first extract the features of patients, diagnoses, and drugs. Then, we employ the sequential generation model to model the sequential decision-making process of the drug regimen. We specify the specific work as follows.

#### 2.2.1 Feature extraction from graph

In clinical practice, most pediatric medicines are dosed according to the patient’s age (23), body height, or body weight (mg/kg) (11). Moreover, treatments also vary according to the patient’s symptom and indication; therefore, diagnostic information is helpful when developing medication regimens (24). As combination drugs are more common in complex prescriptions, they are more likely to cause ADRs. Therefore, drug interactions should also be considered in the rational and appropriate use of drugs. Based on this previous knowledge, we extract the corresponding physiological diagnostic features and drug interaction feature from the medical knowledge graph constructed in the previous phase. Detailed information is provided in the Appendix.

#### 2.2.2 Sequence generation model

In this step, our objective is to predict the rational medication regimen based on the extracted features. One of the intuitive methods is to concatenate the physiology and indication features into a vector and build a regression or classification model to predict the rational medication regimen. However, owing to the considerable variety of the two categories of features, such a direct concatenation of the two heterogeneous features does not perform well, especially when some features play a dominate role in specific medication conditions (25). To address these challenges, we use the sequence generation model to transform the problem into a sequence decision process of the drug regimen, including the medication list and treatment of drug use. Detailed information is provided in the Appendix.

## 3 Experiments

In this section, we evaluate our method with a medical record dataset collected from the MIMIC_III dataset and real-world anonymized prescription big data collected from tertiary hospitals. We first introduce the experiment settings and then present the evaluation results. Finally, we display our analysis results on the visualization platform.

### 3.1 Experiment settings

#### 3.1.1 Dataset

After data cleansing, we obtain a dataset containing 1,084,594 prescriptions with 23,225 patients, 2,393 medicines, and 5,591 diagnoses from the MIMIC_III database, and another dataset containing 230,390 prescriptions with 19,146 patients, 3,782 diagnoses, and 1,198 medicines from tertiary hospitals. The summary of the dataset is shown in Table 1.

**Table 1:** Summary of datasets.

	MIMIC_III	FUJIAN
Data collection period	2001–2008	January 2015–January 2017
# Prescriptions	1,084,594	230,390
# Medicines	2,393	1,198
# Diagnoses	5,591	3,782
# Patients	23,225	19,146

*MIMIC_III dateset*: In this study, we first perform the pre-processing operation of removing invalid patient prescriptions with medical devices, no weight field, and incorrect age statistics. As shown in Table 2, after pre-processing, the dataset contains 11 attributes: (1) patient ID; (2) case number; (3) sex; (4) age, calculated from the patient’s date of birth and admission date, measured in years; (5) Weight, measured in kilograms; (6) diagnosis name; (7) drug ID, National Drug Code for medications; (8) drug name; (9) dosage; (10) dosage unit; and (11) days of administration (the duration of medication usage prescribed by the doctor, calculated from the start and end dates of medication usage, measured in days). The dataset’s characteristics include the presence of multiple hospital admissions for some patients, as evidenced by records 4–5 in Table 2, in which patient “109” has two case numbers: “173633” and “172335”. In addition, the dataset includes instances in which multiple diagnoses were assigned to a patient during a single prescription, with multiple medications prescribed for treatment, as illustrated by records 1–3 in Table 2. For example, for patient “23,” with case number “124321,” the physician assigned two diagnoses, “2252” and “V4581,” and prescribed three medications for treatment: “vancomycin,” “levofloxacin,” and “dexamethasone.”

**Table 2:** Example of the MIMIC_III medical record dataset.

Patient ID	Case Number	Sex	Age	Weight	Diagnosis name	Drug ID	Drug name	Dosage	Dosage unit	Days
23	124321	M	75	66.8	Meningitis	00338355248	Vancomycin	1,000	mg	4
23	124321	M	75	66.8	Meningitis	00045006601	Levofloxacin	750	mg	2
23	124321	M	75	66.8	Meningitis	00054817525	Dexamethasone	4	mg	2
109	173633	F	24	44.9	Hypertensive chronic kidney disease	00172438210	Gabapentin	300	mg	6
109	172335	F	24	66.8	Other primary cardiomyopathies	00182055589	Hydralazine	50	mg	2

*Fujian dataset*: The second historical medical record dataset used in this study is derived from the clinical outpatient data of a tertiary hospital in Fujian Province, China. After pre-processing, this dataset contains a total of 11 attributes, as shown in Table 3. The difference between this dataset and the MIMIC_III dataset lies in the inclusion of prescription numbers and patient heights, while excluding case numbers and drug numbers. As illustrated by the examples in Table 3, it can be observed that the characteristics of this dataset are consistent with those of the MIMIC_III medical record dataset. These characteristics include multiple hospital admissions for patients and instances in which multiple diagnosis information and multiple medications are prescribed for a single hospitalization.

**Table 3:** Example of medical record dataset from Fujian Province.

Patient ID	Prescription number	Sex	Age	Height	Weight	Diagnosis name	Drug name	Dosage	Dosage unit	Days
12**26	d1**2c	M	57	164	58.5	Septicemia	Alfacalcidol	0.25	μg	10
12**26	d1**2c	M	57	164	58.5	Septicemia	Rebamipide Tablets	0.1	g	14
12**26	c0**28	M	57	164	58.5	Nausea and vomiting	Trivitamins ferrous chewable tablets	20	tablet	14
10**26	ae**02	F	79	166	70	Hypertension	Nifedipine controlled release tablets	30.0	mg	14
10**26	ae**02	F	79	166	70	Hyperlipemia	Pitavastatin calcium tablets	2.0	mg	7

#### 3.1.2 Experiment plan

First, we randomly select 80% of the prescriptions collected from the constructed medication knowledge graph for training, and the left 20% for evaluation. Then, we collected 100 problematic prescriptions with inappropriate drug use from small hospitals as a test dataset to evaluate our trained model. Specifically, for each prescription, we use our model to classify whether these prescriptions are an inappropriate use of drugs. We evaluated our model by measuring the proportion of correctly classified prescriptions in terms of the medication sequence list and medication treatment, and using the rational medication regimen to represent the predicted results of both.

#### 3.1.3 Evaluation metrics

To measure the accuracy of the proposed model, we used the Jaccard Similarity Score (Jaccard, as defined in Equation 1), precision (as defined in Equation 2), recall (as defined in Equation 3), and average F1 (as defined in Equation 4), as shown in Table 4. Jaccard is defined as the size of the intersection divided by the size of the union of the ground truth medication regimen $Y_{t}^{\left(k\right)}$ and predicted medication regimen ${\hat{Y}}_{t}^{\left(k\right)}$:

(1) $$\text{Jaccard}=\frac{1}{\sum_{k}^{N}1}\sum_{k}^{N}\frac{\left|Y^{\left(k\right)}⋂{\hat{Y}}^{\left(k\right)}\right|}{\left|Y^{\left(k\right)}\cup {\hat{Y}}^{\left(k\right)}\right|}$$

where N is the number of patients in test set.

**Table 4:** Evaluation metrics overview.

Metric	Description
Jaccard	Measures the similarity between predicted drug prescriptions and actual drug prescriptions.
Precision	Measures the proportion of predicted drug prescriptions correctly identified by the model.
Recall	Measures the percentage of successful identifications by the model in actual drug prescriptions.
F1	Combining the accuracy and recall of the model is a comprehensive metric for evaluating the performance of the model.
DDI Rate	Measures the probability of a drug interaction in a predicted drug sequence.


(2) $$\text{Precision}=\frac{1}{\sum_{k}^{N}1}\sum_{k}^{N}\frac{\left|Y^{\left(k\right)}⋂{\hat{Y}}^{\left(k\right)}\right|}{\left|Y^{\left(k\right)}\right|}$$

(3) $$\text{Recall}=\frac{1}{\sum_{k}^{N}1}\sum_{k}^{N}\frac{\left|Y^{\left(k\right)}⋂{\hat{Y}}^{\left(k\right)}\right|}{\left|{\hat{Y}}^{\left(k\right)}\right|}$$

(4) $$\text{F1}=\frac{2\times \text{\textbackslash{},precision}\times \text{recall}}{\text{\textbackslash{},precision}+\text{recall}}$$

When considering the accuracy of drug prediction, we also need to measure the safety of drug prediction; therefore, to measure medication safety, we define the DDI rate (as defined in Equation 5) to judge the probability of drug interactions in the predicted drug sequence:

(5) $$\text{DDI Rate}=\frac{\sum_{k}^{N}\sum_{i,j}|\{(c_{i},c_{\;j})\in {\hat{Y}}_{t}^{\left(k\right)}|(c_{i},c_{\;j})\in \varepsilon_{d}\}|}{\left|\sum_{k}^{N}\sum_{i,j}1\right|}$$

where the set will count each medication pair $\left(c_{i},c_{\;j}\right)$ in the recommendation set if the pair belongs to the drug interaction adjacency matrix constructed. Here, N is the size of the test dataset.

#### 3.1.4 Baseline methods

We compared our method with several baseline methods with regard to medication regimen prediction and medication regimen adequate evaluation. For medication regimen prediction, we compared our model with several baseline methods as follows.

1. **Bi-LSTM**: this baseline is a sequence-sequence model. At the encoding end, BI-LSTM is used to learn the diagnostic information at the input end, and at the decoding end, ordinary LSTM is used to predict drugs.
2. **GAMENet**: this baseline is a memory-enhancing neural network model that inherits a drug interaction knowledge graph through the graph convolutional network storage module to provide safe and personalized drug combination recommendations.

For appropriate medication evaluation, we compare our model with the following baselines.

1. **Empirical**: This method is based only on the medical experience of professional doctors in tertiary hospitals, without considering the drug contraindication information from existing drug instructions.

### 3.2 Evaluation results

#### 3.2.1 Medication regimen prediction evaluation results

Table 5 shows the rational drug use prediction results from the MIMIC_III dataset using our proposed method as well as the baselines. Results show our proposed method has the highest score among all baselines with respect to Jaccard, precision, recall, and F1. The model we used benefited from the advantages of its structure, which could obtain the relationship between patients’ multiple diagnoses, making it closer to the real doctor’s prescription when making drug predictions.

**Table 5:** The rational medication regimen prediction results of the MIMIC_III dataset.

Methods	Jaccard	Precision	Recall	F1	DDI rate
Bi-LSTM	0.5115	0.6705	0.5697	0.6160	0.1624
GAMENet	0.6517	0.7501	0.6535	0.6985	0.1324
**Ours**	**0.8685**	**0.9555**	**0.8927**	**0.9173**	**0.0867**

Ours indicate the proposed method in this paper and bold values indicate the best performance in the corresponding metric.

Table 6 shows the rational drug use regimen prediction results for the outpatient medical record dataset using our proposed method as well as the baselines. Results show our proposed method achieves the best performance compared with other baseline methods. As this dataset is different from MIMIC_III, no authoritative drug classification has been performed, and drugs with therapeutic equivalence exist in this dataset as multiple drugs. Therefore, we chose to provide three alternative elements for each element generated by the sequence model. It is deemed to be the correct prediction when the actual use of the drug appears in the three alternatives. The calculation formula of its evaluation index is shown in Equation 6.

(6) $$\text{Precision}=\frac{1}{\sum_{k}^{N}1}\sum_{k}^{N}\sum_{i}^{M}\frac{\left|Y_{i}^{\left(k\right)}\in {\hat{Y}}_{i}^{\left(k\right)}\right|}{M}$$

where $M=\min(|Y^{\left(k\right)}|,|{\hat{Y}}^{\left(k\right)}|)$.

**Table 6:** The prediction results of the outpatient medical record dataset.

Methods	Precision
Bi-LSTM	0.4355
GAMENet	0.6355
**Ours**	**0.7769**

Bold values indicate the best performance in the corresponding metric, and Ours indicate the proposed method in this paper.

Table 7 clearly shows the predicted results of rational drug use of the model in the Fujian province dataset. The actual prescriptions shown on the left of the table below are two drugs taken by a boy for allergic rhinitis, mycoplasma infection, and bronchitis. On the right are the recommendations for drug therapy provided by our model. It can be found that the model in this paper can accurately cover the real prescription after providing three alternatives, and most of the other alternatives provided are also drugs for the treatment of respiratory diseases such as rhinitis. It indicates that the model in this study can obtain the drug recommendations of actual doctors according to patient diagnosis and other characteristics.

**Table 7:** Drug recommendation cases in the Fujian Province dataset.

Real prescription	Predict prescription
Ganan mixture	Ganan mixture,
	Josamycinpropionate granules,
	Mometasone furoate aque
Loratadine tablets	Montelukast sodium oral granules,
	Loratadine tablets,
	Ambroterol oral solution

In addition, we conducted two comparative experiments to determine whether the addition of patients’ physiological and DDI features could improve the effect of our model. Table 8 shows the accuracy rate of drug recommendation is improved after the introduction of physiological characteristics in our model. Table 9 shows that after the introduction of DDI features in our model, although the accuracy of model prediction is slightly sacrificed, the probability of adverse drug interactions caused by recommended drugs is reduced. Therefore, we can adjust the weight proportion of DDI characteristics to meet the application requirements.

**Table 8:** The influence of physiological features.

	Jaccard	Precision	Recall	F1
No physical features	0.8556	0.9546	0.8727	0.9069
Have physical features	**0.8627**	**0.9564**	**0.8855**	**0.9131**

Bold values indicate the best performance in the corresponding metric.

**Table 9:** The influence of DDI features.

	DDI rate	Jaccard
No DDI features	0.0906	0.8239
Have DDI features	**0.0837**	**0.8167**

Bold values indicate the best performance in the corresponding metric.

#### 3.2.2 Medication regimen appropriateness evaluation results

In this study, we use the trained models as classifiers to judge the appropriateness of prescriptions in small hospitals. Table 10 shows the average accuracy scores of medicine use appropriate evaluation using the proposed method and the baselines. We can see that the proposed method achieves the best performance with regard to evaluation accuracy scores. Specifically, the baseline method *Empirical* attempts to evaluate the appropriate use of drugs based only on the experience of drug use of professional doctors in tertiary hospitals, which results in some combination drugs with adverse reactions being misjudged. In summary, the proposed method integrates the two heterogeneous information to model sequential patterns and therefore improves the accuracy of evaluation. The calculation formula of its evaluation index is shown in Equation 7.

(7) $$\text{Accuracy}=\frac{1}{\sum_{k}^{N}1}\sum_{k}^{N}\sum_{i}^{M}\lfloor \frac{\left|Y_{i}^{\left(k\right)}\in {\hat{Y}}_{i}^{\left(k\right)}\right|}{M}\rfloor ⊙{\hat{T}}_{i}$$

where $M=\min(|Y^{\left(k\right)}|,|{\hat{Y}}^{\left(k\right)}|)$, and $\hat{T}$ represents the rationality marker of prescriptions in small hospital.

**Table 10:** The appropriate evaluation of prescription in small hospitals.

Methods	Accuracy
Empirical	83%
Ours	**89%**

Bold values indicate the best performance in the corresponding metric.

### 3.3 Clinical appropriate medication evaluation system

**Figure 5:**
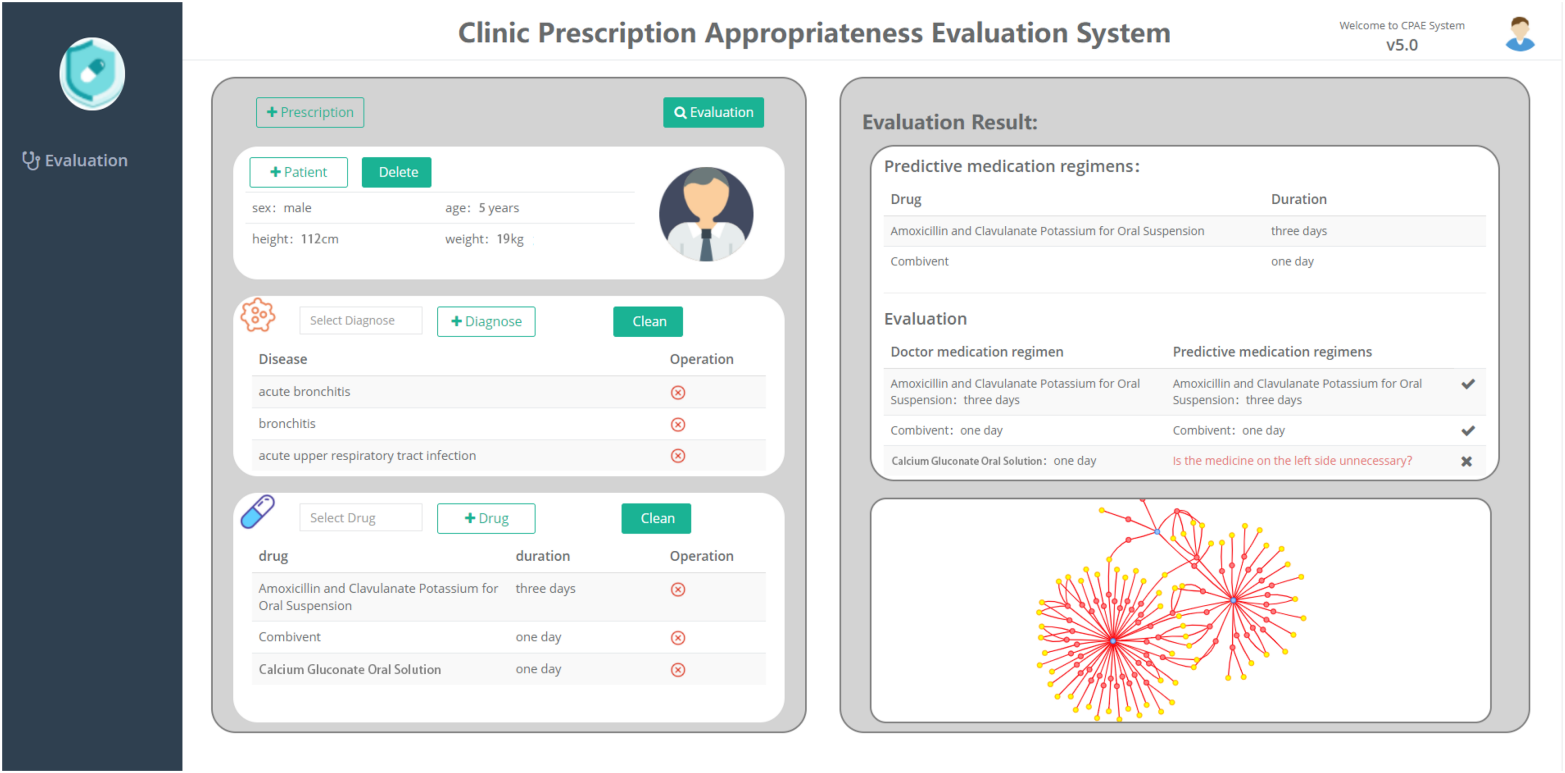
Clinical prescription appropriateness evaluation system.

To demonstrate the work in this paper more clearly, we have built a platform for the appropriateness of rational drug use and applied it in a small hospital to evaluate the appropriateness of doctors’ prescriptions. As shown in Figure 5, this platform is mainly divided into two parts, among which the left view is divided into three subgraphs (patient–disease–drug), and the right view shows the evaluation results. In the left frame, you can first fill in patient information, diagnostic information, and drug information. Then, click the button to evaluate for the appropriateness of prescribing. Finally, the predicted medication results are displayed on the right side of the frame, with a tabulated comparison of the doctor’s medication regimen and the predicted medication regimen. At the same time, the entity relationship involved with the disease in the medication graph would be visualized below the evaluation results, which would be convenient for doctors and pharmacists to further review and modify prescriptions.

### 3.4 Case study

We conduct a case study of one prescription randomly selected from 100 problematic prescriptions of inappropriate medications in small hospitals. As shown in Figure 5, in the input prescription, the patient is a male, has a height of 112 cm, has a weight of 19 kg, and is 5 years old. The patient’s diagnosed symptoms were acute bronchitis, bronchitis, and an acute upper respiratory tract infection. The prescribed medicines were amoxicillin and clavulanate potassium for oral suspension, Combivent, calcium gluconate oral solution, and the corresponding treatment courses are 3, 1, and 1 day(s). Based on our proposed framework, the predicted medication regimen was amoxicillin and clavulanate potassium for oral suspension for 3 days and Combivent for 1 day. After evaluation, the system would provide default color labels and red labels to represent the consistent medication regimen and inconsistent medication regimen, respectively. The red label in the picture indicated whether calcium gluconate oral solution are unnecessary drugs.

## 4 Conclusion

In this study, we investigate one of the key problems in rational medication, i.e., the evaluation of appropriate medication use. We propose a framework of appropriate drug use based on medical association and big data to accurately predict the medication regimen by leveraging prescription big data and medical text data. Specifically, a medication knowledge graph is first constructed based on historical prescription big data and medical text data from tertiary hospitals. Then, we employ a GMM for physiological features, BERT for diagnostics, and graph convolutions for drug interactions, yielding accurate medication regimens. Our approach surpasses baselines in predicting regimens and detecting appropriate medications, and was validated on MIMIC_III and real-world prescription data from tertiary hospitals.

One of the limitations of this study is the *feature selection*. There might be other indication or physiology features that could be associated with medication regimens and used as predictive features for example. For example, for adolescents, the probability of developing corresponding diseases during adolescence can also be considered in the prediction model to improve the prediction accuracy in teenagers. We are currently working with hospitals to retrieve richer information related to prescription datasets, such as picture archiving and communication systems and inspection results from laboratory information systems, which we believe will provide useful and important features for drug regimen prediction.

In the future, we plan to extend our work in the following directions. First, we plan to involve more data sources from other hospital information systems, especially data from clinical laboratories, to investigate more relevant factors of doctor medication. Second, we plan to investigate the reasons for the wrong medication sequence list, including overtreatment or undertreatment by model overfitting, and then leverage the knowledge to improve our predictive models. Third, we plan to integrate our method with the existing clinical decision support systems to provide dosing recommendations for doctors and pharmacists in small clinics.

## Figures and Tables

**Figure A1:**
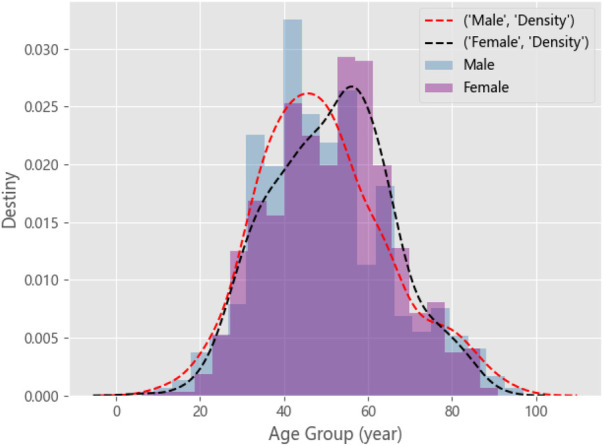
Distribution of patients with chronic gastritis.

**Figure A2:**
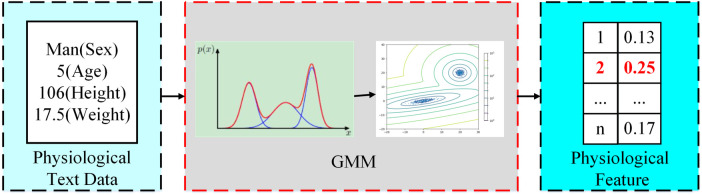
The framework of physiological feature extraction.

**Figure A3:**
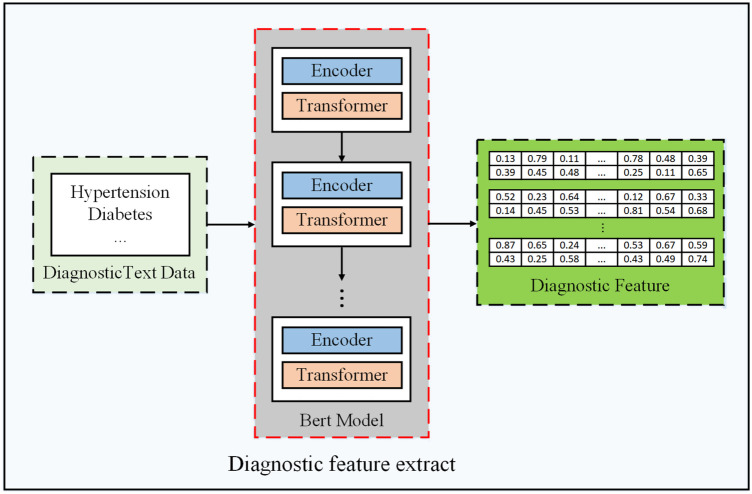
Framework of diagnostic feature extraction.

**Figure A4:**
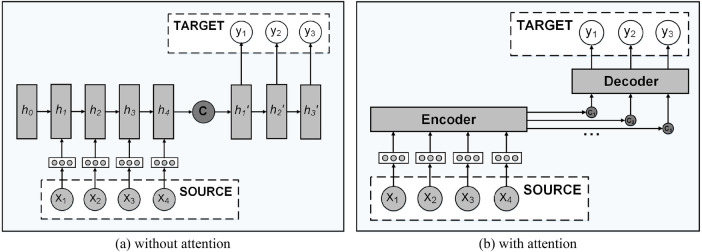
The framework of diagnostic feature extraction.

**Figure A5:**
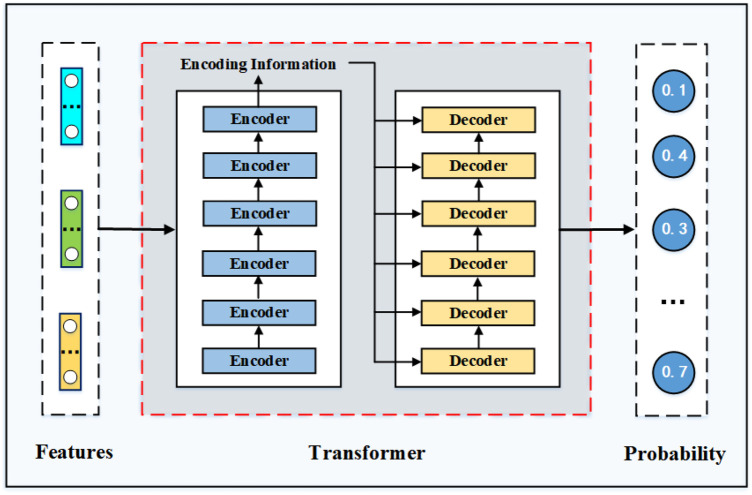
The transformer model framework.

## Data Availability

The raw data supporting the conclusions of this article will be made available by the authors, without undue reservation.

## Appendix

### 1 Graph node and edge construction

*Clinical experience edges.* We first construct the clinical experience edge set $R_{a}$ based on the triples extracted from prescription big data. Specifically, the edge set $R_{a}$ of graph G are defined as follows: for the triples extracted from the prescription record, we set up two nodes and assign a directed edge between the corresponding nodes in graph G. The triples include “Patient-Have-Prescription,” “Prescription-Diagnose-Disease,” “Prescription-Prescribe-Drug,” “Patient-Have-Disease,” and “Patient-Use-Drug.”

*Medical knowledge edges.* The second step is to construct medical knowledge edges $R_{b}$ based on the triples extracted from the drug instructions. Specifically, the edge set $R_{b}$ of graph G are defined as follows: for the triples extracted from drug instructions, we set up two nodes and assign a directed edge between the corresponding nodes in graph G. The triples include “Drug-Ingredient-Drug,” “Drug-Indication-Disease,” “Drug-Contraindication-Patient,” “Drug-Contraindication-Disease,” “Drug-Interaction-Drug,” and “Drug-Contraindication-Drug.”

### 2 Feature extraction from graph

*Physiological feature extraction.* In this step, our objective is to extract patients’ physiological information from historical prescription big data as the features of rational drug use. Physiology metrics of patients, such as sex, age group, body weight, and body height, are usually the most important considerations in clinical medication calculation. Although the combination of these factors provides the greatest accuracy in calculating medication regiments, simple digital groupings of different age groups, heights, or weights could lead to excessive discretion in the population sample. Therefore, we group patient populations as a physiological feature by modeling physiological information.

Owing to differences in gender, height, weight, and other physiological characteristics, the distribution of prescription data may be composed of N Gauss. For example, patients with chronic gastritis. By plotting the distribution of patients of different ages in male and female genders, as shown in Figure A1, we can observe that there are approximately two component kernels in the distribution. The component kernals were contributed by male patients aged approximately 50 and female patients aged approximately 60. Therefore, we can use a mixed Gaussian distribution to fit all patient prescription data. We use the BMI to represent height and weight for each prescription data. First, we estimate the optimal number of component cores n for the patient’s prescription data using the Akaike information criterion (AIC) (26) and Bayesian information criterion (BIC) (27, 28). The number of component cores is optimal when the AIC and BIC are as small as possible. Then, we calculate the probability distribution of the patient in each component kernel. Finally, we take the group with the highest probability as physiological characteristics, as shown in the Figure A2. The probability density function of the GMM is given by Equation (A1):

(A1) $$p(x)=\sum_{k=1}^{K}\pi_{k}N(x|u_{k},\Sigma_{k})$$

where X is the age distribution of prescriptions, K is the number of sub-Gaussian models in GMMs, and $\pi_{k}$ is the mixture coefficient, which is the probability that each observation data belongs to the kth submodel. The $N(x|u_{k},\Sigma_{k})$ is the distribution function, $u_{k}$ is the expectation, and $\Sigma_{k}$ is the covariance of the kth component in the mixed model. The above variables satisfy Equations (A2) and (A3):

(A2) $$\sum_{k=1}^{K}\pi_{k}=1,$$

(A3) $$0<\pi_{k}<1$$

With the EM algorithm (expectation-maximization algorithm) (29), we can iteratively calculate the parameters in the GMM: ($\pi_{k},x_{k},\Sigma_{k}$ ). In short, the EM algorithm has two steps. The first step is E (expectation), which updates the implicit variable. The second step is M (maximization), which is used to update the parameters of each Gaussian distribution in the GMM. Then, the above two steps are repeated until the iteration termination condition is reached.

*Diagnostic feature extraction.* In this step, our objective is to extract diagnostic sequence information as diagnostic features of the patients. A simpler approach to represent diagnostic information is to digitize the diagnosis. However, the diagnostic text representation is rich in the semantic information of words, such as text similarity. Therefore, considering that word vectors are rich in more semantic features, we choose to represent diagnostic features by transforming text into word vectors through a BERT pre-trained word model (20), as shown in Figure A3. We elaborate the details as follows.

When sending a diagnostic text into BERT, it will encode the text into an input vector, the length of which is always 512. For an input vector, it is composed of three embedding features: (1) WordPiece (30), (2) position embedding, and (3) segment embedding. Furthermore, as shown in Figure A3, its framework consists of the multi-layer transformers proposed by Vaswani et al. (31). Transformers realize a series of encoding and decoding to transform an input text into a possible predicted result. Finally, the diagnostic text is converted to tokens by BERT, and a word vector at the corresponding position of each token is printed. We take out the results of the penultimate hidden layer and use the results of all vector mean pooling as diagnostic features.

*Drug interaction feature extraction.* In this step, our objective is to establish drug interaction relationships from the medication knowledge graph as another feature. It can be found that the main form of drug interaction relation stored in the medication knowledge graph is pair, and it is more effective to use the graph structure to represent the drug interaction relation. Therefore, we used such a pair relationship to generate the drug interaction matrix A to represent the drug interaction graph. However, the interaction diagram exists as a two-dimensional matrix, whereas physiological and diagnostic features exist as one-dimensional vectors. Therefore, we need to transform the characteristics of drug interaction so that it can be spliced with the other two characteristics as the input of a reasonable drug recommendation model. We elaborate on the details as follows.

The size of the drug interaction matrix is N×N, where N is the size of the drug set. For the MIMIC_III dataset, we first select two drugs in the drug set randomly, assuming that the coded values are i,j. Then, we map its ATC4 code to the CID classification. Finally, we use the CID code as the keyword to search the drug interaction database for adverse drug interaction risks in these two categories. We iterate through all drug pairs in the database to generate an adjacency matrix of the final drug interaction diagram, which reflects the currently known drug combination contraindications and can reduce the number of treatment options that produce ADRs when a drug is recommended. For the medical record data of Fujian Province, we also randomly select two drugs in the drug set, and then search in the medication knowledge graph with the keyword of drug composition for whether these two drugs have an adverse drug interaction risk. If there is, the corresponding element of the marker matrix is 1, otherwise it is 0. The simplest way to convert a two-dimensional matrix to a one-dimensional vector is to compress the matrix. However, such compression destroys the connection between nodes, making it impossible for the model to learn the complete drug interaction relationship. Therefore, we employ the graph neural network method to embed the two-dimensional drug interaction characteristics into the one-dimensional space. The specific transformation process is described as follows.

Like the convolutional neural network in image vision, the GCN (21) is used for feature extraction. Therefore, we use the basic graph convolution network to construct a simple graph neural network, which maps the graph node representation to the low-dimensional vector space while preserving the topology and node information of the graph. A graph neural network with two GCN layers is established in this study, where A is the graph structure and X is the matrix representation of the graph. The GCN layer compresses the hidden representation of each node by aggregating the feature information from the node neighborhood, and after the feature aggregation, non-linear permutation, such as ReLU, is applied to the generated output. Through the stacking of multiple layers of the GCN, the final hidden representation of each node in the diagram obtains information from subsequent neighborhoods. Finally, we connect it to a fully connected network to obtain a one-dimensional vector output. Through the transformation of the graph neural network, we obtain the one-dimensional representation of the characteristics of drug interactions.

### 3 Sequence generation model

First, the symbols are defined, with X representing the diagnostic space and Y representing the drug treatment space. $R=\{(X_{1},Y_{1}),(X_{2},Y_{2}),\ldots ,(X_{k},Y_{k})\}$ is a set of prescription records, $X_{k}\subseteq X$ is a diagnostic sequence, and $X_{k}=\{X_{1}^{k},X_{2}^{k},\ldots ,X_{\left|Y_{k}\right|}^{k}\}$, $Y_{k}\subseteq Y$ is a sequence of medication regimen, $Y_{k}=\{y_{1}^{k},y_{2}^{k},\ldots ,y_{\left|Y_{k}\right|}^{k}\}$. $\left|X_{k}\right|$ and $\left|Y_{k}\right|$ are the sequence lengths of $X_{k}$ and $Y_{k}$, respectively. There is no explicit mapping of the corresponding elements between diagnostic sequence $X_{k}$ and drug sequence $Y_{k}$. To avoid confusion, if there is no ambiguity, we omit k in the symbol. The purpose of drug prediction is to select the best sequence of medication regimen $Y_{k}$ among all drugs Y based on the diagnostic sequence x. Therefore, the model in this study should have the ability to learn to map any diagnostic sequence to a corresponding medication regimen sequence, which requires the model in this study to learn not only the relationship between drugs and diagnosis but also the relationship between drugs and drugs. Therefore, we used the popular transformer approach in NLP to generate drug sequences. We will take a brief look at one of the key mechanisms in the transformer model and the overall architecture.

*Attention mechanism.* The transformer is based on the attention mechanism; therefore, before introducing the overall framework of the transformer model, we first introduce the role of the attention mechanism. The sequence generation model transformer is composed of an encoder module and a decoding module. As shown in Figure A4(a), the encoder compresses the information expressed by the input sequence into a fixed-length semantic vector, and then the decoder decodes the information based on this semantic vector and generates the target sequence one by one. It means that elements at any point in the input sequence are equally important to the current target element. This method of learning input sequence cannot express the position information of the sequence, and if the sequence is too long, the decoding effect of the fixed semantic vector will be poor, because it is easy to lose the information contained in the sequence. To solve the above problems, Luong et al. (32) proposed the attention mechanism in 2015. As shown in Figure A4B, the mechanism generates an independent semantic vector for each element in the output sequence, which could express the different importance of each input sequence to the decoded target element. The semantic vector $C_{i}$ is the weighted sum of the elements in the input sequence, as described in Equations (A4) and (A5):

(A4) $$C_{i}=\sum_{j=1}^{L_{x}}\alpha_{ij}h_{j}$$

(A5) $$\alpha_{ij}=\frac{exp(e_{ij})}{\sum_{k=1}^{T_{x}}exp(e_{ik})}$$

(A6) $$e_{ij}=a(s_{i-1},h_{j})$$

where $L_{x}$ is the length of the input sequence, α is the distribution of attention, $\alpha_{ij}$ represents the importance of the element in the input sequence to the element that determines the output sequence, and $h_{j}$ represents the implicit state of the jth element in the input sequence. In fact, α is a similarity measure, which is calculated according to the correlation between the jth element in the input sequence and the ith element in the output sequence. As shown in A6, $e_{ij}$ is obtained from the output $S_{i-1}$ of the hidden layer at the time of i−1 in the decoder and the correlation degree of the hidden state $h_{j}$ corresponding to each element in the encoder. There are many methods to calculate the similarity between $s_{i-1}$ and $h_{j}$. In this study, we adopt the dot product method as shown in A7 to calculate the similarity.

(A7)
$$a(h_{t},{\bar{h}}_{s})=h_{t}^{T}{\bar{h}}_{s}$$

The above attention mechanism focuses on the relationship between the input sequence and output sequence, which can help us obtain more information when we model the relationship between drug and prescription. However, the relationship between elements in the input and output sequences is not taken into account. To solve this problem, another new attention mechanism, self-attention (33), has been proposed and widely used. Compared with the use of various cyclic neural networks that require longer information accumulation, the model with the introduction of the self-attention mechanism cannot only obtain the dependency relationship between two elements that are far apart, but also obtain the dependency relationship between the internal elements of the sequence more easily. In addition, the self-attention mechanism also improves the parallel computing capability of the model, greatly reducing the training time of the model. The transformer model used in this study is also based on the self-attention mechanism.

In the self-attention mechanism, the input sequence will be represented in the form of key value pairs, and then the input sequence with N elements will be represented as $\left(K,V)=[(k_{1},v_{1}),(k_{2},v_{2}),\ldots ,(k_{N},v_{N})\right]$, where the key value is used to compute the attention distribution α and the value is used to compute the semantic vector. The output sequence will be represented as N queries; therefore, the semantic vector computation problem can be considered as an addressing operation. We use query to find the key=query element in the input sequence, and the value obtained is the semantic vector or called attention. In particular, the self-attention mechanism can be thought of as soft addressing. Instead of looking only for elements with key values that are equal to the query value, a weighted sum is applied to all values to calculate the final attention. The weight of each value is determined by calculating the similarity between the query and each key. Therefore, the formula for calculating attention is shown in Equation (A8):

(A8) $$\text{Attention}(Q,K,V)=\text{softmax}\left(\frac{QK^{T}}{\sqrt{d_{k}}}\right)V$$

where $Q\in R^{n\times d_{k}}$ , $K\in R^{m\times d_{k}}$, and $V\in R^{m\times d_{v}}$; therefore, attention is a $n\times d_{v}$ matrix. Different from the general attention mechanism, the self-attention mechanism also performs a division operation when calculating the attention distribution coefficient to avoid the similarity value calculated by the inner product method being too large, which results in 0 or 1 being generated when the softmax function is used for normalization, losing the meaning of soft addressing.

*Overall framework of transformer.* The transformer model is a machine translation model proposed by Google in 2017 Vaswani et al. (31). As shown in Figure A5, the transformer model is mainly composed of an encoder module and a decoder module. Each module is composed of several encoder and decoder layers, and the number of stacked layers can be adjusted according to the difficulty of tasks. This model abandons the traditional RNN or CNN architecture and adopts the structure of a full self-attention stack, which achieves excellent performance in NLP tasks. In this study, we build a two-layer transformer model to carry out the task of rational drug recommendation.

There are three kinds of attention in transformer, namely, self-attention in the encoder, self-attention in the decoder, and attention between the encoder and decoder. To capture all the spatial information in the input sequence, the attention calculation method in transformer is improved on the basis of the previous introduction and the concept of multi-head is introduced. It is mainly used to project query, key, and value to different spaces for H times through linear transformation, and then h self-attention matrix is obtained through calculation. As the feed-forward layer can only receive one matrix, we finally splice the h matrices and multiply by a weight matrix to generate the final attention matrix. When self-attention between the input and output sequences is calculated, set Q=K=V, and the specific calculation process is shown in Equation (A8) and (A9):

(A9) $$\text{MultiHead}(Q,K,V)=\text{Concat}({\text{head}}_{1},\ldots ,{\text{head}}_{h})W^{0}$$

(A10) $${\text{head}}_{i}=\text{Attention}(QW_{i}^{Q},KW_{i}^{K},VW_{i}^{V})$$

As we can see from Figure A5, the decoder structure is similar to that of the encoder, with the difference that the decoder has two attention layers. The decoder’s goal in decoding is to give a probability distribution for the first output element. Therefore, we need to calculate the attention value of the last decoder layer in the decoder module. The input to this attention layer consists of two parts: the output of the last encoder module and the output of the first decoder module in the decoder module. Therefore, in calculating the attention of the decoder layer, the value K, V in A9 comes from the encoder and Q comes from the decoder. Decoder decoding is different from the encoder in that it can compute in parallel because it needs to use the output of the previous decoder layer as a query; therefore, the decoder is used one by one to generate the elements of the output sequence. In the model training process, the decoder layer uses real values; therefore, the mask method should be used to calculate the self-attention between output sequences to ensure that the current model cannot obtain more information than the current location.

Based on the transformer model, as shown in Figure A5, we join the features together as the input sequence. After a multistep process of encoding and decoding, we select the element with the highest probability in the probability distribution as the prediction result for the current position, until we either reach the end of the generated identifier or the maximum sequence length. The resulting sequence is used as the model’s recommendation for the current patient. Next, we will verify the effectiveness of the transformer model in the prediction of rational drug use through several comparative experiments.
